# Encoding of Situations in the Vocal Repertoire of Piglets (*Sus scrofa*): A Comparison of Discrete and Graded Classifications

**DOI:** 10.1371/journal.pone.0071841

**Published:** 2013-08-13

**Authors:** Céline Tallet, Pavel Linhart, Richard Policht, Kurt Hammerschmidt, Petr Šimeček, Petra Kratinova, Marek Špinka

**Affiliations:** 1 Institute of Animal Science, Ethology Department, Prague, Czechia; 2 INRA, UMR1348 PEGASE, Saint-Gilles, France; 3 Agrocampus Rennes, UMR1348 PEGASE, Rennes, France; 4 Cognitive Ethology Lab, German Primate Center, Göttingen, Germany; University of Sussex, United Kingdom

## Abstract

Two important questions in bioacoustics are whether vocal repertoires of animals are graded or discrete and how the vocal expressions are linked to the context of emission. Here we address these questions in an ungulate species. The vocal repertoire of young domestic pigs, *Sus scrofa*, was quantitatively described based on 1513 calls recorded in 11 situations. We described the acoustic quality of calls with 8 acoustic parameters. Based on these parameters, the k-means clustering method showed a possibility to distinguish either two or five clusters although the call types are rather blurred than strictly discrete. The division of the vocal repertoire of piglets into two call types has previously been used in many experimental studies into pig acoustic communication and the five call types correspond well to previously published partial repertoires in specific situations. Clear links exist between the type of situation, its putative valence, and the vocal expression in that situation. These links can be described adequately both with a set of quantitative acoustic variables and through categorisation into call types. The information about the situation of emission of the calls is encoded through five call types almost as accurately as through the full quantitative description.

## Introduction

The question as to whether vocalisations in mammals vary continuously in a graded system, or are clustered in a set of distinct call types [1], [2] is becoming more and more important in the current upsurge (i.e. publications quadrupled within the last 10 years) of animal vocalisation research. This is because the continuity-discontinuity question of the acoustic repertoire has a direct bearing on whether and how animals themselves classify vocalisations [3]. Studies have been carried out that support the existence of both discontinuous and continuous repertoires in mammals. For instance, in rhesus monkeys (*Macaca mulatta*) evidence for natural classification is strong as there are five acoustically distinct food calls and the monkeys consider some of the classes closer to each referentially in spite of their acoustic differences [4], [5]. Some authors base their conclusions on the assumption that the repertoire is made of call types [6] and limit their analyses to describing the different call types. However there is the example of Barbary macaques (*Macaca sylvanus*) which have a continuous acoustic variance across differently sounding calls indicating a graded link with the internal state of the caller [7]. A recent study [3] also suggests that Campbell’s monkeys (*Cercopithecus campbelli*) use both discrete and graded variations at different levels of urgency. One intriguing possibility is that vocal repertoire in some species may be more or less continuous at the senders’ end but distinctly categorial at the perceivers’ end. In other words, the senders encode information about their state or their assessment of the situation through analogue mapping of the acoustic structure of their calls but the receivers cut this continuum into disjoint perceptual categories of “call types“ [8], [9]. For instance, humans have a natural tendency to categorize colours into distinct categories although such categorisation is “objectively baseless“, given the smooth continuum of wavelengths. Thus in addition to describing the vocal emissions, it is also important to take into account the situation and determine if this vocal expression is situation-typical. This has often been described qualitatively [6], [10] but rarely quantitatively. We may expect that there can be similarities in vocal expressions in different situations when the emotional state is close and dissimilarities in vocal expressions between situations which are different [11], [12]. Finding characteristics (i.e. proportion of call types) typical to situations of emission will allow us to progress in our understanding of the link between vocal expression and emotions, and will be useful for welfare assessment too [13].

For an in-depth understanding of an acoustic communication system in a species, a detailed quantitative description of the repertoire, including an assessment of acoustic (dis)continuity, is a pre-requisite. It also provides a common basis for the scientists who work on this topic, to which they can report to make comparative studies. It is also necessary for cross-specific comparisons investigating the evolution of call structures [2], [6]. It may also help to address specific hypotheses like the “social function hypothesis” which assumes that the more complex a social species is, the more diverse its vocal repertoire should be [14], [15]. Moreover, a full qualitative description of the vocal repertoire of a species, including the situations in which the different-sounding calls are used, gives the opportunity to establish how the emotional state of the sender and/or the functional significance of the situation are encoded in the calls [11], [16]. However, we still lack high-quality descriptions for vocal repertoires even for common and intensely investigated species such as the domestic pig.

The domestic pig is a vocal species that produces diverse calls in different situations. The richness of the pig vocal repertoire is reflected in the variety of labels that have been used for pig calls. For instance, piglets “scream” in painful situations [17], “bark” when they are surprised [18] or when they play [19], “croak” during naso-nasal contacts with mother [20] and “grunt” when briefly isolated [21]. These verbal labels may lead us to believe that the vocal repertoire of pigs is made of qualitatively discrete categories of calls. On the other hand, the sub-classification that some authors used like short/long-grunt [22], or the use of mixed call types like squeal-grunts and grunt-squeals [23], [24] suggests that the acoustic structure of pig calls may vary continuously across a quantitative acoustic spectrum. Pig acoustic communication has been under intense scrutiny in recent 15 years with research focusing on issues such as the function of specific types of calls [25], on the emotional content [26], [27] and on the potential use of vocalizations as indicators of pig welfare [13]. Some studies included objective description of the variation in acoustic structure of calls but were restricted to specific situations such as isolation, castration, human approach test or suckling [21], [22], [28], [29]. A study that would structurally describe and compare pig vocalizations across a large variety of situations and provide a comprehensive pig vocal repertoire has not been accomplished yet.

In this study, we aimed at describing the variability of the vocal repertoire of piglets before weaning and determining if it is possible to classify the calls into discrete types or whether calls vary continuously within the acoustic space. Secondly, we analysed how the vocal expression varies across a range of 11 different situations. Thirdly, we examined how accurately the situation of the piglet could be determined from the vocal quality of the calls and if the accuracy based on the putative call types is comparable to accuracy based on quantitative acoustic parameters. Finally, we quantified the link between the vocal quality and the degree of negativity of the situation of emission, as evaluated by experts.

## Methods

### Ethic Statements

This study received approval for animal use and care from the Institutional Animal Care and Use Committee of the Institute of Animal Science and was conducted in accordance with approval number 44248/2007–17210 of the Czech Central Committee for Protection of Animals.

### Animals

We studied (Large White×Landrace) × (Duroc×Pietrain) piglets between 6 and 15 days of age. The pigs were born at the experimental farm of the Institute of Animal Science in Prague-Uhříněves (Czechia). The animals were reared in a standard way in a farrowing room where several lactating sows with their litters were housed and could hear and partially see each other. Each litter was housed in a concrete floored, straw-bedded (2.2 m×2.0 m) pen, which included the heated nest area (1.0 m×0.5 m) for the piglets. The lactating sow was housed in an ellipsoid-farrowing crate (2.2 m×1.4 m) positioned in the pen.

### Vocalisations Recording

Recordings were made in 11 different contexts of emission (i.e. situations, Table 1). The 11 situations could be grouped into three main types according to their biological significance: life threatening situations (castration, crushing, holding piglet in arms and fighting for teat); nursing situations (before nursing, after nursing and missed nursing); and general social situations (huddling, isolation, reunion with mother and surprise). In some situations, only some piglets did vocalise. This was the case before and after nursing (only one or two piglets of a litter generally vocalise at each nursing), and also when the piglets were held by a human (some piglets remained silent and motionless). Both males and females were recorded and their overall number was balanced (except for castration). Before the acoustical analyses, we selected the recordings with a low background noise. All together 1513 calls (data are available on request by contacting the corresponding author) were analysed from 93 recordings made on 84 different piglets belonging to 34 litters (Table 1). We identified 34 males and 28 females. Identity of the remaining 22 piglets was not determined because we did not want to disturb the behaviour of the animals (e.g., during fighting, huddling or nursing). Each piglet was recorded only in a single situation with the exception of 9 piglets that were recorded in two different situations. The duration of the situations varied naturally. For instance “surprise” situation was logically very short lasting (1s) and duration of “nursing situations”, “fighting”, “huddling” and “reunion” depended on the naturally occurring sequence of mother-young interactions (2–15s). Similarly, duration of “castration” situation depended on the procedure (30s). For all these situations, we analysed the whole natural sequence, without cutting. In those situations where we could control the duration of recording, we tried to use the duration that corresponds to the natural situation. For instance, “crushing“ lasted 30 s because it is the time in which a sow should respond to the piglet calls to prevent crushing, isolation lasted 10 minutes to be perceived as getting lost (last 100s were analyzed).

**Table 1:** Description of the situations of recordings and data collected.

Abbreviation	situation	description of the situation	Numberof calls	Numberof piglets	Numberof different litters
Life threatening situations					
CA	Castration*	during routinely performed castration. The sow and litter remained in theirlactating pens, and male piglets were transferred one by one to the isolated room.A stockperson hold the piglet between his legs, while the veterinarian performedthe castration surgery in a standard way without anaesthesia. Calls were recordedboth during the “holding” phase and the “cutting” phase.	171	7	5
CR	Crushing*	The situation when a sow traps a piglet under her body was simulatedthrough restraining a laterally lying piglet by both hands against the floor.For detailed description of the methods see [51].	159	10	10
AR	Arms of a person*	holding by a stockperson. The experimenter took the piglet, and held it for1 min in her arms, the mouth directed toward the microphone.	209	6	4
FI	Fighting	when piglets fought for teats during nursing [51].	115	5	4
Nursing situations					
BN	Before nursing	when a piglet came at the head of the sow before nursing [20].	61	5	5
AN	After nursing	when a piglet came at the head of the sow after nursing [20].	128	14	9
MN	Missed nursing	when a piglet was struggling to get to teats for nursing because it wasbehind the back of a lying sow.	70	6	4
General social situations					
HU	Huddling	when huddling together with littermates. Huddling consists of piglets lying pressed tightly together, often in two or three layers one over another.	81	10	3
IS	Isolation*	during a period of separation and isolation from the sow and the littermates.The piglet was taken in the arms of the experimenter and put intoa small pen (50 cm×50 cm×50 cm) for 10 minutes.	222	5	5
RE	Reunion*	After the isolation, when the piglet was put back to the sow and littermatesand vocalized next to the head of the sow (situation described in [65]).	279	15	5
SU	Surprised*	when an isolated piglet was surprised by the arrival of a person. After theisolation period, the experimenter suddenly appeared above thepen silently (previously used by [18]).	18	10	6

* situations that were provoked by a human being.

Five of the situations were spontaneous and six were provoked (Table 1). The spontaneous situations and the Reunion situation were recorded in an 2.9×2.8 m room that contained one farrowing pen with equipment identical to the pens in the farrowing room. The room was separated from the rest of the pigsty by solid brick walls and solid doors that significantly dampened the noise from the pigsty and thus enabled a high signal-to-noise ratio of the records to be obtained. On the day preceding the recordings, the animals were transferred to this room so that they could become habituated to the new situation. The piglets were marked with a non-toxic livestock marker for individual recognition. The experimenter (C.T.) sat next to the animals for three hours with the recording equipment so that the sow and piglets could get used to her presence. If after this period the sow remained reactive to the experimenter’s movements, then she was excluded from the experiment. During the recordings, a microphone (Sennheiser® ME67, Sennheiser®, Wennebostel, Germany) was held by the experimenter about 50 cm from the animal’s mouth. The microphone was linked to a sound recorder (Marantz® PMD671, Marantz® Europe, The Netherlands). The experimenter stayed with each litter for a total of 10 hours. For the provoked situations, individual piglets were taken to another room. This room was in a prefabricated building in which no animals were kept and that was situated 10 m away from the pigsty. In this room, the same recording equipment was used as in the preceding one, but the microphone was hanging on a stand. The room contained a testing pen (50 cm×50 cm×50 cm) made visually isolated by solid walls.

### Ranking of Situations According to the Degree of Negativity

To measure the possible link between the emotional state of the piglets and their vocal expression, we assigned the 11 situations to a quantitative emotional valence [30] using expert opinion. Using an email questionnaire, we asked 28 pig behaviour experts from 14 countries to rank the 11 situations according to the valence of the situation for the piglet. Valence was defined as the degree of negativity of the situation for the piglet. Rank 1 denoted the most negative situation for the piglet and rank 11 the least negative/most positive situation for the piglet. We then took mean negativity rank for each situation as the standard for the situation.

### Preparation of the Sound Files and Acoustic Analyses

Sounds were processed and analysed in Avisoft SASLab PRO 5.1.21 (Raimund Specht, Avisoft Bioacoustics, Berlin, 2011). The sampling frequency of recordings was 44 100 Hz. Only calls with high signal-to-noise ratio, non-overlapped with other sounds were used for analyses, therefore no filters were applied. We used Hamming window, 1024 FFT-length, and window overlap of 87.5%, to generate spectrograms with frequency grid resolution equal to 43 Hz and time grid resolution equal to c. a. 3 ms. Length of the analysis window was 23 ms. Individual calls were first identified and cut manually by spectrogram and oscillogram inspection. We described calls with a number of different spectral, temporal, frequency and amplitude modulation and tonal quality parameters (Table S1). These were derived from spectra of entire calls or from 11 measurement windows (each measurement window corresponded to single FFT analysis window; window length = 23 ms) spaced in regular intervals within a call (beginning of the call and then in intervals equal to call length/10). The first and the last measurement windows were excluded and we used only the nine measurement windows located well within the call for further analyses. We used q50 (the frequency that divides the spectrum into two frequency intervals of equal energy) rather than peak frequency to calculate the frequency modulation measurements, as peak frequency could vary greatly between steps in noisy calls and q50 provided better tracking of frequency modulation judged by the spectrograms. Due to difficulties in measuring F0 in a substantial proportion of calls (noisy calls), we could not have the fundamental frequency for all the calls and decided not to use it in the analyses.

As we looked for distinctive classes we focused only on the parameters that showed multimodal distributions in the subsequent analyses [24], [31]. We further checked for pair-wise correlations between variables. Only one of the variables from a pair with a high correlation coefficient (>0.9) has been retained in the analysis. We preferred to exclude highly correlated variables rather than reduce the dimensionality of the dataset by Principal Component Analysis for example, to ensure easier and more straightforward interpretability of the results. The 8 variables finally used for cluster analysis were:

- peak frequency; frequency with the highest amplitude; derived from spectrum of entire call (pf),
- frequency that divides the spectrum into two frequency intervals of equal energy; derived from spectrum of entire call; amplitude is integrated over the whole frequency range of spectrum and split by frequency into equal halves (q50),
- mean Wiener entropy of the call; ratio of the geometric mean to the arithmetic mean of the spectrum; as Wiener entropy intrinsically increases with bandwidth of the sound, we scaled the value reported by SASLab to q50 (ent). A low entropy corresponds to a tonal sound, while a high entropy corresponds to a noisy sound; derived from spectrum of entire call,
- q50 at the beginning of the call (at the first of 9 measurement windows; q50start),
- q50 at the end of the call (at the last of 9 measurement windows; q50end),
- minimum value of q50 among the 9 measurement windows within the call (q50 min),
- duration (dur),
- relative location of q50max within the call (number of measurement window with the maximum q50 value; q50maxloc).

Because acoustic variables were on different scales they were centred and standardized (so that the mean of each variable was 0 and its variance was 1) prior to cluster and Linear Discriminant Analysis (LDA).

### Statistical Analyses

All statistical analyses were carried out in R [32].

We used the k-means clustering method to see whether the calls would fit into several call types. We had no prior expectations about number of call types (i. e. number of clusters). Therefore we ran cluster analyses assuming 2–15 clusters and subsequently judged the validity of obtained cluster solutions based on the EtaK and PreK values [7]; EtaK = 1– (WSS_K_/WSS_1_) and PreK = 1– (WSS_K_/WSS_K-1_). EtaK reflects the relative reduction in within-cluster variance for a solution with a given number of clusters (WSS_K_) compared to variance of the unpartitioned dataset (1-cluster solution, WSS_1_). PreK reflects relative improvement in the reduction of variance for a given solution (WSS_K_) compared to previous solution (WSS_K-1_). From the conclusions of the cluster analysis, we calculated the parameters of the central call of each cluster. Then, to have an illustration of each cluster, we reported the typical call of each cluster with the shortest Euclidian distance from this centre.

LDA and MANOVA were used to classify the calls into situations based on the eight selected acoustic variables or based on call types determined by the cluster analysis and to test for differences in acoustic variables between situations. Situation was the grouping variable. Independent variables were eight acoustic call parameters or, in the case of classification based on call types, call type membership which was coded as 1 or 0 for each call type (e. g. assuming three call types – A, B, and C – call type “C” would be coded by the three variables as follows: A = 0, B = 0, C = 1). LDA can be used on nominal variables, mainly as a black-box classification algorithm, even though the LDA assumptions are often violated in such cases. We checked carefully that the classification gave meaningful results (i. e. the call was assigned into the situation with highest proportion of corresponding call type in case of single call; the “averaged” call was assigned into the situation with the most similar call type proportions in case of multiple calls available for classification, see below). Results of LDA were cross-validated using the holdout method [33]. Holdout method was performed as follows. The whole dataset was first split into two halves. One half of calls was used for determination of discriminant function (calibration dataset). Discriminant function was then applied to the other half calls (validation dataset) to obtain the classification of calls into situations. To evaluate classification success, we compared the number of obtained correct classifications with the distribution of correct classifications by chance on permuted dataset (1000 permutations) [34]. This approach has been suggested for groups with unequal sample sizes [35]. As several calls from a single piglet were often used and different piglets and piglet numbers were recorded in each situation, we used pDFA [36] to see whether the results remain significant when individuality is accounted for. We used agglomerative hierarchical cluster analysis to investigate acoustic similarity between situations. This method successively links the most similar objects into larger groups. Average values of acoustic parameters or call type proportions were used as input data. Euclidean distances were used as measurement of distance between the pair of situations and Ward’s criterion was the linkage criterion. Ward’s criterion minimizes the within cluster variation.

We wanted to describe the distribution of the call types according to the situations. To test if the distribution of call types among situations was random, we used Pearson chi-square test or chi-square simulated by Monte Carlo permutation test (when expected contingency table was lower than 5). We then applied a rule of thumb: Pearson residuals >2 and Pearson residuals >4, to interpret associations of call types with situations.

To determine the probability of classifying the calls (based on the 8 parameters or on the call types) into the proper situation, we used a simulation method. The simulation was based on the increasing number of “accessible” calls and was cross-validated as “leave sample out” procedure. We randomly selected a sample of one to 20 calls for each situation (as there were only 18 surprise calls we did not include them in this analysis) and averaged their acoustic parameters (for classification based on acoustic variables) or computed the proportions of call types within a sample (for classification based on call types). Thus we obtained 10 averaged samples; one for each situation. These 10 samples were subsequently classified based on the discrimination function derived from the rest of the calls. This procedure was repeated 10 times resulting in 100 samples (10 from each situation) being classified before the overall percentage of correct classifications was computed. The whole simulation was repeated 20 times to obtain the smooth confidence intervals for correct classification percentages.

We used Spearman rank correlation to see whether the acoustic parameters of calls averaged within each situation with the estimated valence rank of the situation (as evaluated by experts).

We tested the influence of the estimated negativity rank of the situations on the probability that the emitted call would belong to a particular call type with a logistic linear model. The probability that the emitted call would belong to a particular call type was calculated as a proportion of a given call type within all calls recorded in a given situation. Because the dispersion parameter was significantly larger than 1, we used quasi-binomial likelihood model (family “quasibinomial” in R model specifications) that compensates for over-dispersion.

We controlled for false discovery rate [37] when multiple similar hypotheses were tested instead of controlling for family wise error rate (e. g. Bonferroni correction) as recently suggested [38], [39].

## Results

### Description of the Vocal Expression

#### Variation in the vocal expression

**Figure 1:**
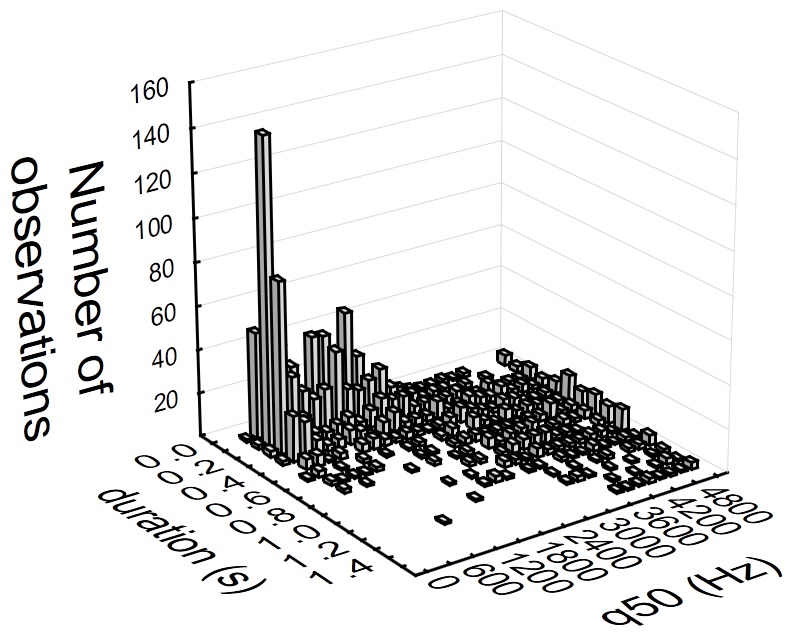
Bivariate histogram of the number of calls. Histogram is built up according to the duration and the median of the distribution of frequency amplitudes (q50) of the calls. To construct the histogram, duration bin was set to 0.1 s and q50 bin was set to 200 Hz. Values higher than 99 percentile for both variables were all grouped into the last bin.

The 1513 calls varied hugely in acoustic quality. For instance, the range of durations was between 0.034 s and 2.138 s, and the q50 varied from 340 Hz to 7350 Hz (Figure 1). Acoustic variables used for analysis were bimodal or multimodal, and hence some cluster structure was apparent when inspecting univariate or bivariate histograms (see Figure 1 for an example).

#### Possibility of classifying calls into clusters

**Figure 2:**
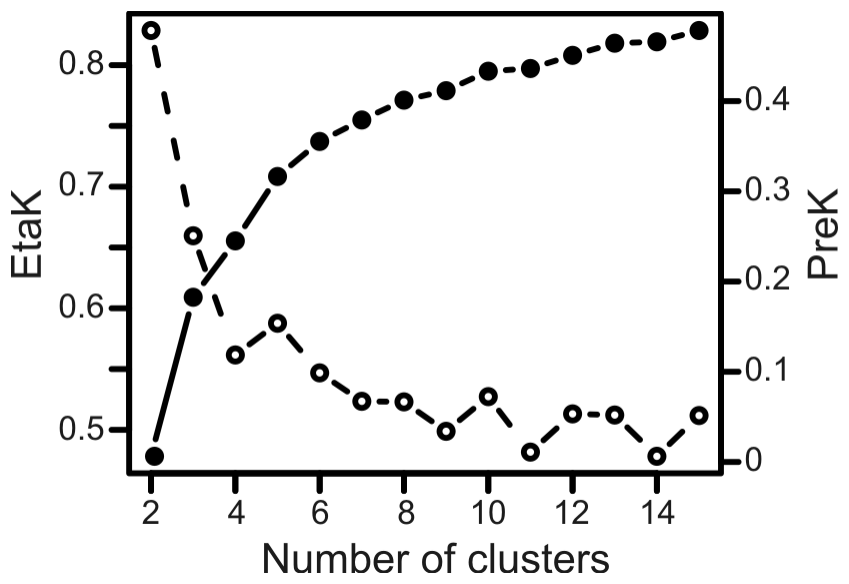
Evolution of the two indices EtaK and PreK according to the different cluster solutions. EtaK (right y-axis) is represented by filled circles and full lines. PreK (left y-axis) is represented by open circles and dashed lines.

From the course of the two indices EtaK and PreK (Figure 2), apart from the two-cluster solution, the no-cluster solution is clearly superior to any other. The trace of EtaK did not show any global or local peaks. The 2-cluster solution already leads to 50 per cent reduction of within cluster sum of squares. EtaK further increased steeply to the 5-cluster solution. Classifying the calls into more than 5 clusters did not lead to substantial EtaK index increments. The 2-cluster solution had the highest PreK value. There was a local peak in PreK at the 5-cluster solution and minor peaks at 10-, 12-, and 15-cluster solutions. We further considered only 2- and 5-cluster solutions for classification of calls into call types.

**Figure 3:**
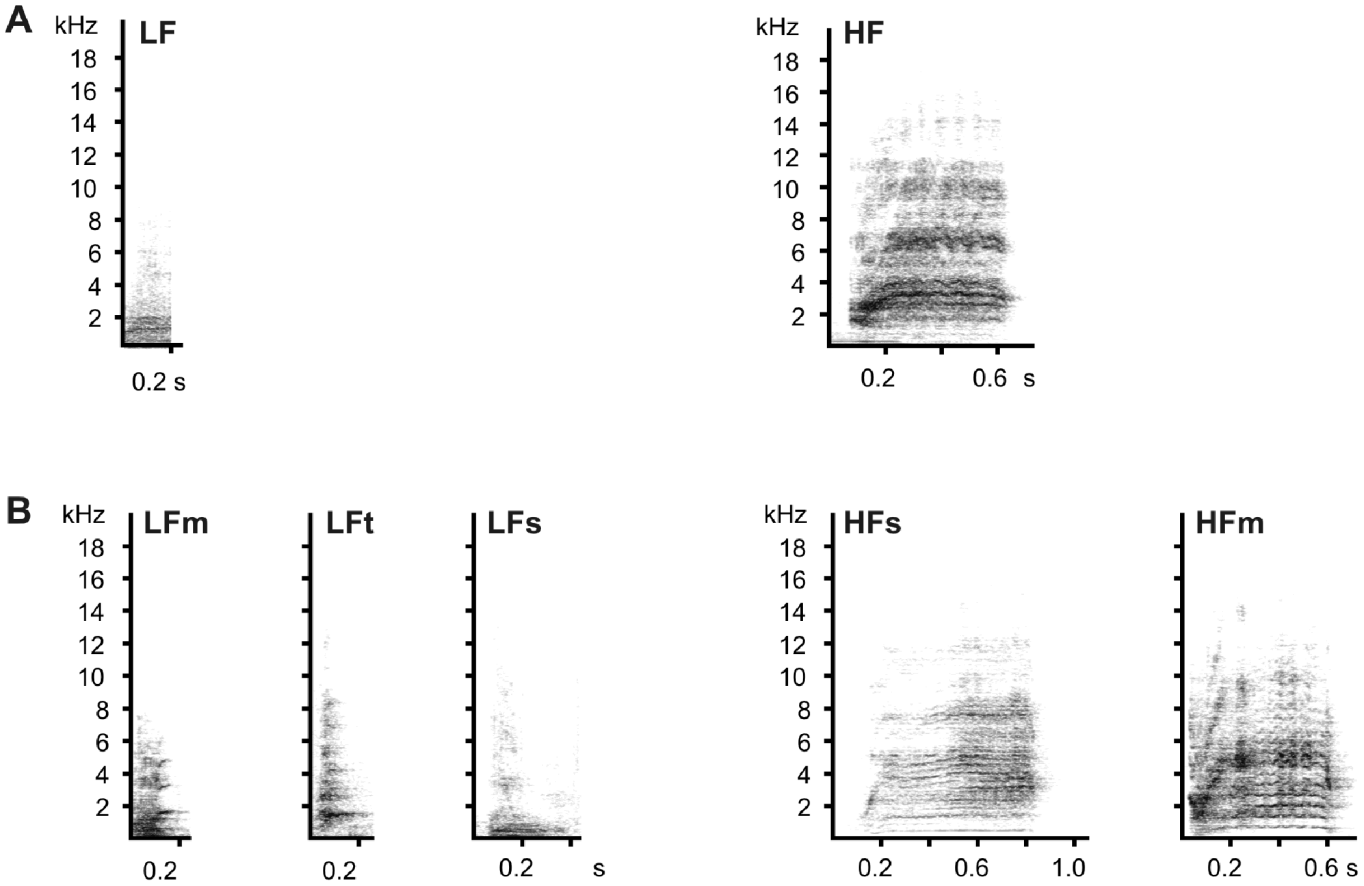
Spectrograms of representative calls for each cluster A. in 2-cluster solution and B. 5-cluster solution.

**Figure 4:**
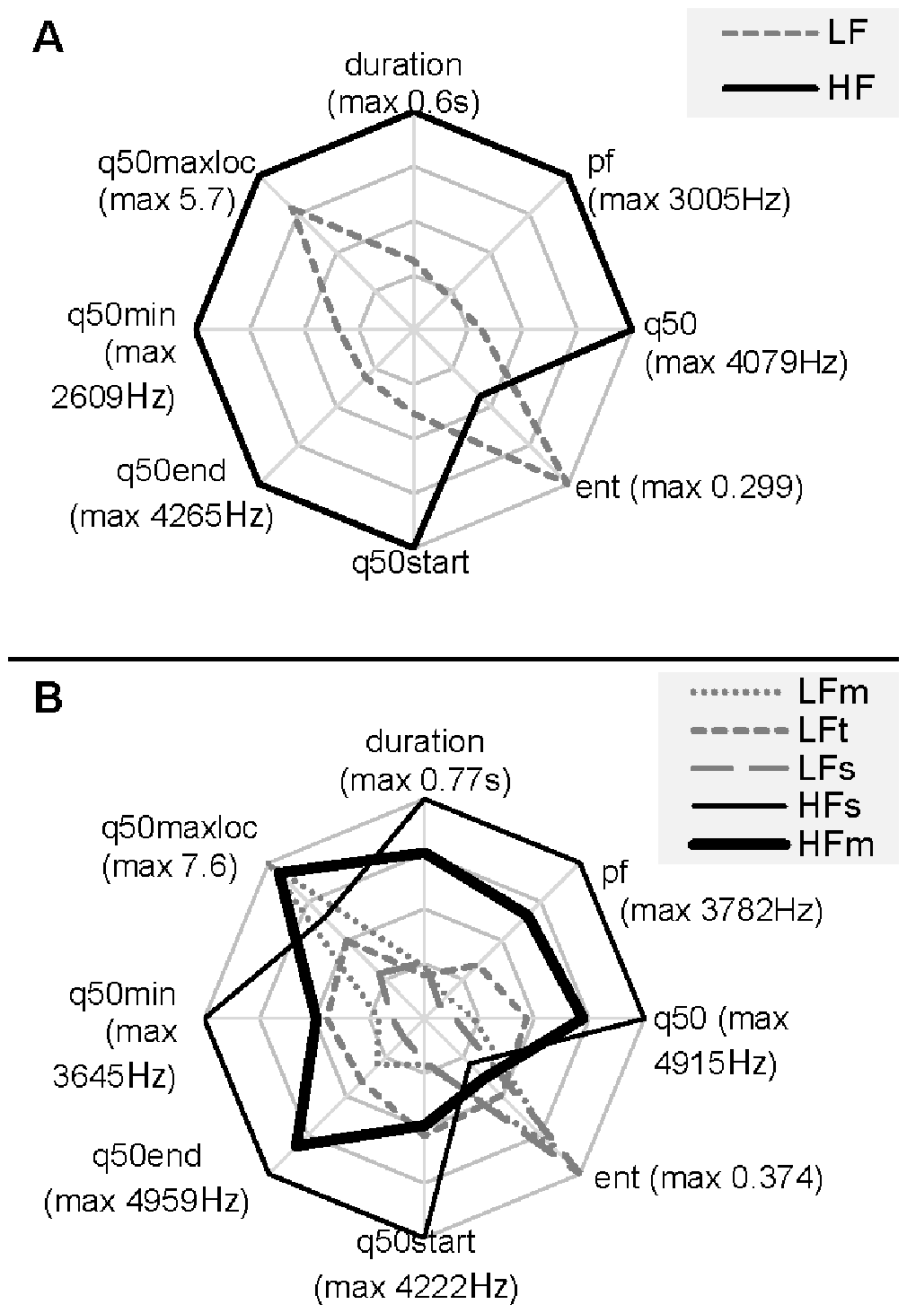
Polar plot representing the mean acoustic properties of the clusters for A. 2-cluster and B. 5-cluster solutions. Each polar axis ranges from 0 to the maximum of each parameter. Maximum values are noted in brackets.

To show the specificity of each call type, the spectrograms of representative calls from each cluster (calls with the shortest Euclidean distance from the centre of each cluster) are shown in Figure 3. Sixty-nine per cent of the calls were included in cluster 1 corresponding to calls shorter in durations and lower in frequencies than calls in cluster 2 (Figure 4a, Table S2). We will further refer to cluster 1 as low frequency calls (LF) and cluster 2 as high frequency calls (HF) (Figure 4a, Table S2). Naming corresponds to the fact that the two pitch variables (pf and q50) had the highest loadings when call clusters were subjected to discriminant function analysis (Table S3).

These two clusters apparently split into three (LF) and two (HF) subclasses, respectively, in the 5-cluster solution. All calls of the first and third cluster of the 5-cluster solution and 83% of calls of the second cluster were LF calls of the 2-cluster solution. All calls of the fourth cluster and 90% of the calls of the fifth cluster were HF calls of the 2-cluster solution. Consequently, clusters of the 5-cluster solution were named LFm, LFt, LFs, HFs and HFm (where subscripts are referring to characteristic features for the particular cluster – see below) (Figure 4b, Table S2). Loadings of each variable for the first two discriminant functions are given in Table S3.

Low frequency modulated calls (LFm, 21% of all calls) are low calls with increasing frequency. Low frequency tonal calls (LFt, 25% of all calls) have the highest frequency, the lowest entropy among LF calls and are not very frequency modulated. Low frequency stable calls (LFs, 26% of all calls) are the lowest among LF calls and were not very frequency modulated. High frequency stable calls (HFs, 12% of all calls) have the highest pitch and are the longest calls, but are not very frequency modulated. High frequency modulated calls (HFm, 16% of all calls) increase greatly in frequency between the start and the end of the calls.

### Differences in Vocal Expression between Situations

#### Differences in the acoustic parameters between situations

**Figure 5:**
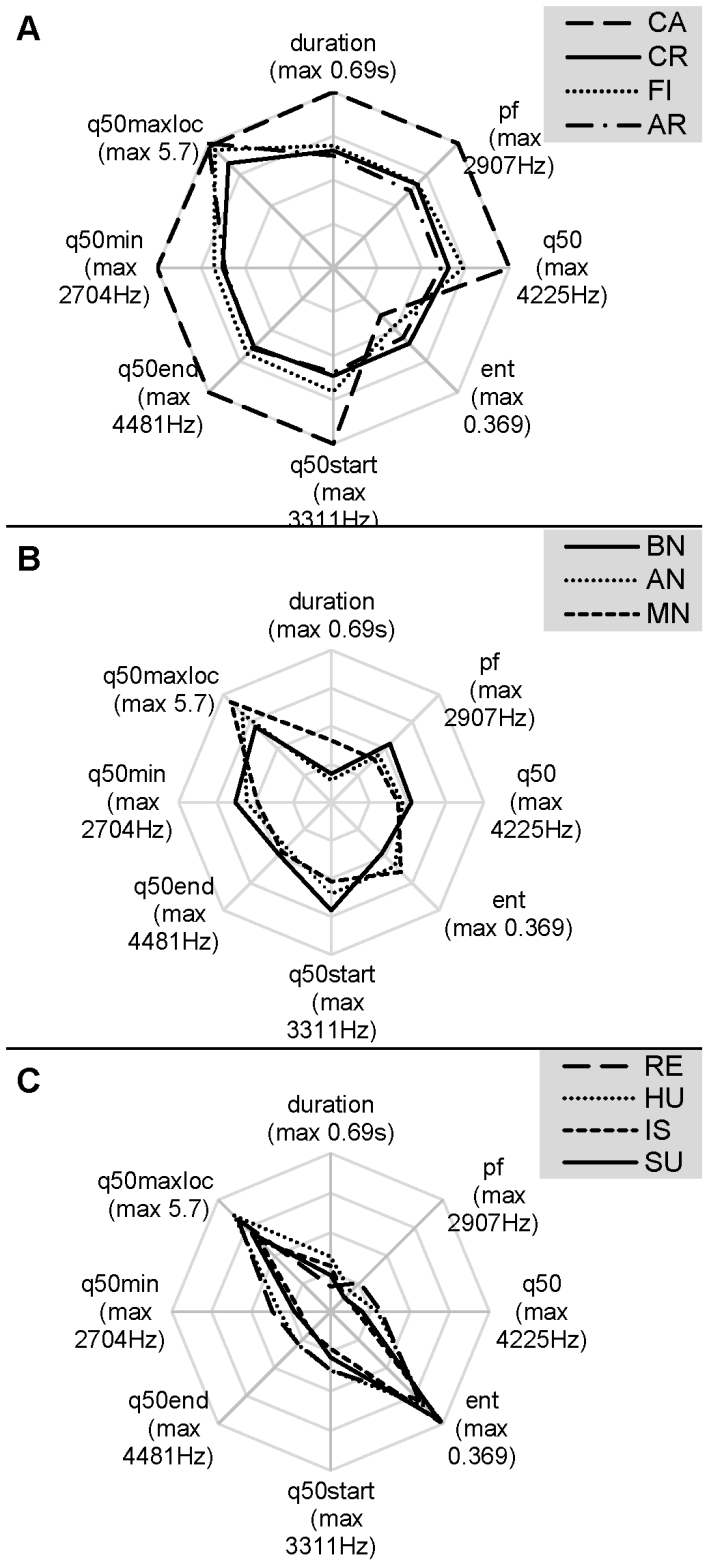
Polar plot representing the mean acoustic properties of the calls in each situation. We grouped A. negative situations: Castration, Crushing, Fighting and Arms, B. nursing-related situations: Before nursing, After nursing, Missed nursing, and C. other situations: Reunion, Huddling, Isolation and Surprise. Each polar axis ranges from 0 to the maximal mean of each parameter (values in brackets).

Multivariate test revealed that, based on the eight variables, average acoustic structure of calls differed among situations (MANOVA: *Wilks λ* = 0.29, *F*_10,742_ = 12.8, *P<*0.001, Figure 5). Castration was clearly different from all the other situations, producing the longest, highest-pitched and most tonal calls (with lowest entropy) (Figure 5a). The other three negative situations for the piglets (Figure 5a) were characterised by somewhat lower, but still quite high values (between 50 and 75% of the castration values) of duration and pitch, and moderate entropy. Situations around nursing (Figure 5b) provoked the shortest and quite noisy calls, with start and minimum pitch similar to the three negative situations, but lower mean and end pitch values. However, the missed nursing calls were acoustically somewhere in the middle between the other nursing situations and the last category depicted in Figure 5c. This last category of situations (reunion, huddling, isolation and surprise) incited very low-pitched and most noisy vocalisations that were somewhat longer than nursing calls (Figure 5c). There was little between-situation variation in q50maxloc.

**Figure 6:**
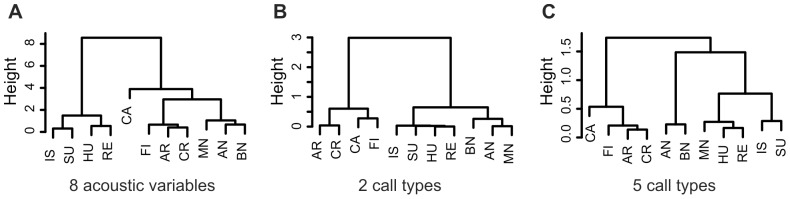
Dendrograms of situation similarity according to A. acoustic parameters, B. two call types and C. five call types. Hierarchical clustering with Ward’s method was used to construct the dendrograms. Height represents cophenetic distance (dissimilarity) between the situations. Distances are estimated by Ward’s criterion for group fusion. AN = after nursing, AR = in the arms of a person, BN = before nursing, CA = castration, CR = crushing, FI = fighting, HU = huddling, IS = isolation, MN = missed nursing, RE = reunion with the sow and litter, SU = surprise.

The acoustic (dis)similarity of calls emitted in different situations was evaluated by hierarchical cluster analysis. Results are illustrated by a dendrogram (Figure 6a). Calls from situations that have similar biological significance (i.e. life threatening situations, nursing situations, and other social situations) were acoustically similar to each other. The only deviation from this pattern is that castration was not in the same cluster as the other three life threatening situations but rather on the next level of branching.

#### Differences in the call type proportions between situations

**Figure 7:**
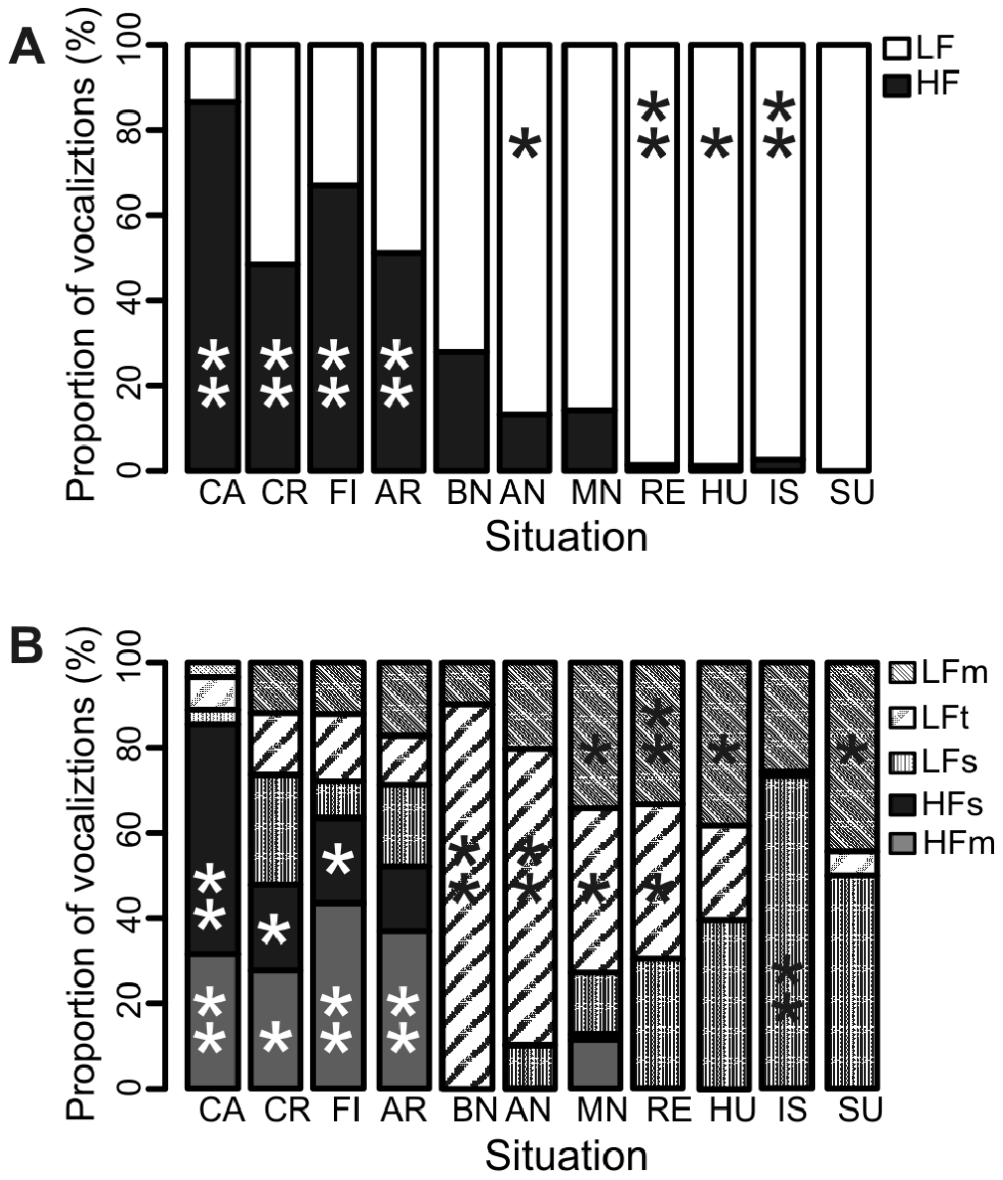
Proportion of call types emitted in each of the situation. A. 2-cluster solution and B. 5-cluster solution. Asterisks (* or **) mean that the number of calls is higher than the expected number for a random distribution; i.e. *: Pearson residuals >2 and **: Pearson residuals >4.

The four negative situations had the highest proportion of HF calls; the mother-related situations had moderate levels of HF calls. Huddling, isolation, reunion and surprise mostly induced exclusively LF calls (Figure 7a). When 5 call types were considered (Figure 7b), the negative situations were the only ones with HFs calls and also had the highest proportion of HFm calls. The mother-related situations had the highest proportions of LFt calls and the remaining four situations provoked the highest use of LFs calls. Reunion was in-between with high proportions of LFt and LFs. Distribution of call types among situations was not random (2-cluster solution: Chi-square test: χ^2^ = 649, df = 10, *P*<0.001; 5-cluster solution: Chi-square from Monte Carlo permutation test with 2000 replicates: *χ* ^2^ = 1296.5, *P*<0.001, Figure 7). Whatever the solution, LF calls were particularly strongly associated with reunion and isolation while HF calls were particularly strongly associated with the negative situations (*Pearson residuals* >4). On a finer scale, LFm were strongly associated with reunion, LFt with after- and before nursing situations, LFs with isolation, HFs with castration, and HFm with holding in arms, castration, and fighting (Figure 7b).

The dendrograms (Figure 6b and 6c) reveal that in situations of similar biological significance (i.e. in four life-threatening situations, in three nursing situations, in four other social situations), a similar mixture of call types is used by the piglets. Especially when just 2 call types are distinguished (Figure 6b), the match between gross biological significance of the situation and the proportion of high-pitched calls is perfect. For the 5 call types solution (Figure 6c), there is one exception as the Missed Nursing situation adheres to the other social situations rather than to the remaining two nursing situations.

### Identifying the Proper Situation from Vocal Expression

#### Identifying situation based on acoustic parameters of one or more calls

**Figure 8:**
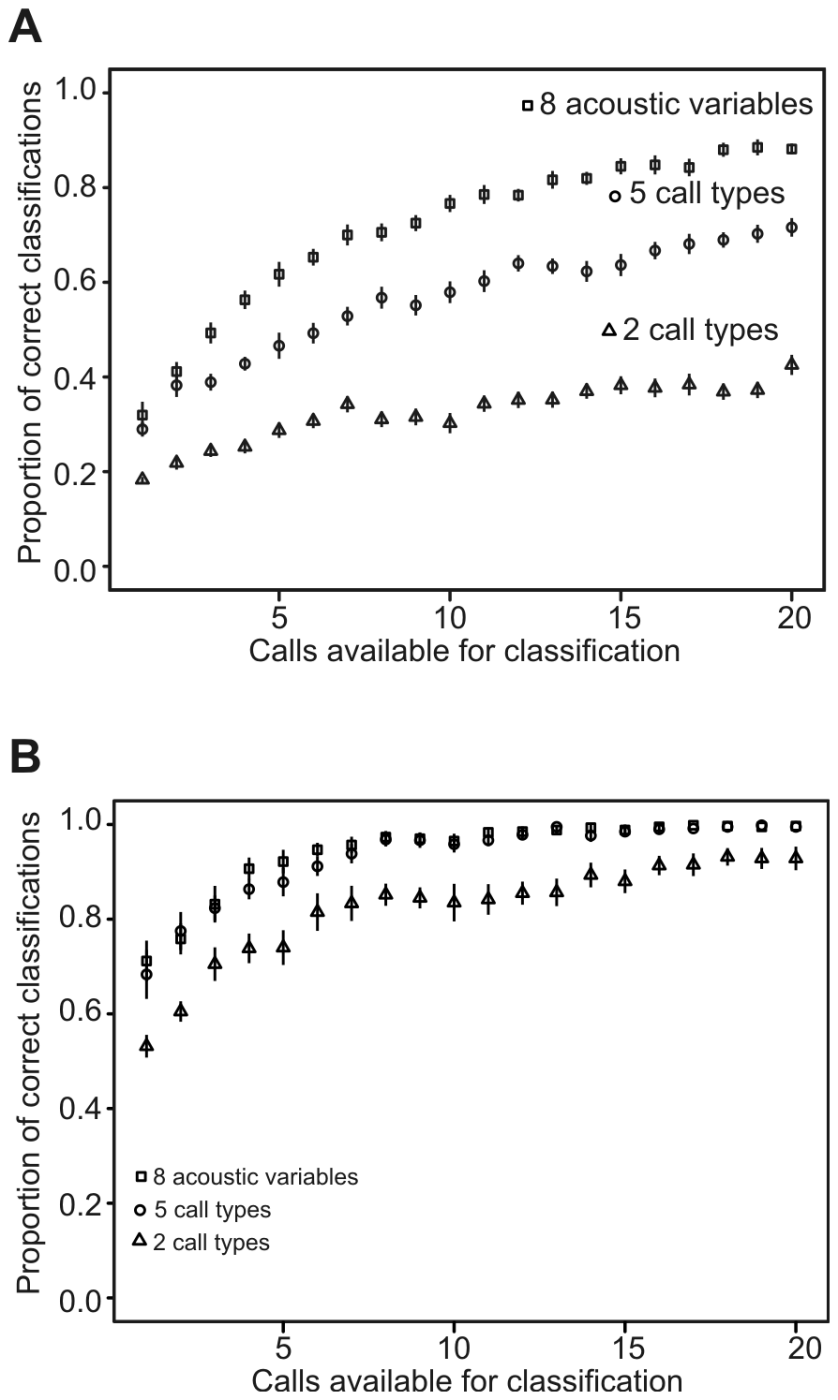
Simulation of average overall classification success of calls to situations depending on number of calls available for classification. A. Classification into one of the 11 situations; B. classification in the gross biological type of situation (life threat/nursing/other). The situation of the piglet was identified from the call(s) based either on 8 acoustic variables (square) or on types of the calls – either 2 call types (triangle) or 5 call types (circle). Mean proportion of correct classifications and 95% Confidence Interval for the mean from 20 repeated simulations are displayed.

The LDA (holdout cross-validation) based on acoustic parameters of a single call classified 28% of the calls into the correct situation (out of 11 possible), which is significantly above chance level (*P<*0.001; expected correct assignments based on chance = 7.3%). The four most easily recognised situations were Castration (62% correctly classified), Before Nursing (55% correctly classified), Isolation (53% correctly classified) and After Nursing (34% correctly classified). When identity of caller was accounted for, results remained significant (pDFA: *P*<0.001). Simulation (leave-sample-out cross-validation) showed that with increasing number of calls from the respective situation, the success at identifying the correct situation increased from 32% at 1 call up to 88% at 20 calls (Figure 8a).

The simulation also demonstrated that the gross biological significance of the situation could be very reliably assessed from the acoustic parameters of the calls. If the classification did not aim at identifying the exact situation but only at distinguishing whether the situation was life-threatening (situations CA, CR, AR, FI), nursing-related (BN, AN, MN) or generally social (HU, IS, RE, SU), then already one call was sufficient to achieve a 76% classification success and a 100% correct classification was reached with eight calls (Figure 8b).

#### Identifying situation based on call types of one or more calls

The single-call LDA (holdout cross-validation) based on 2 call types classified correctly 12.6% of the calls to appropriate situation which was not significantly different from the random classification (*P* = 0.080, expected correct assignments based on chance = 5.8%; pDFA: *P*>0.1). The classification success improved only slowly with increasing number of calls reaching 43% of correct situation identification with 20 calls (Figure 8a). Note that in the case of only one call available for classification, call type was simply attributed to the situation in which the particular call type prevailed. LDA (holdout cross-validation) based on 5 call types correctly classified 24.7% of single calls to the appropriate situation, which differed significantly from the random classification (*P<*0.001, expected correct assignments based on chance = 6.6%; pDFA: *P*<0.001). The classification success for one and two calls did not differ from that attained when using 8 acoustic variables for classification (*P>*0.1). When taking more calls into account, classification success increased at a pace that was slower but more similar to the classification made with eight acoustic variables than to the one with 2 call types. Seventy-two per cent of correct identifications were obtained with 20 calls (Figure 8a).

Distinguishing the gross biological significance of the situation was as highly successful when it was based on call types as when it was based on acoustic parameters (Figure 8b). Knowing the call type of just one call allowed a 68% correct judgment whether the situation was life-threatening, nursing-related or generally social. In addition a mere eight calls were sufficient to achieve a 100% correct assessment.

### Vocal Expression According to the Degree of Negativity of the Situation

#### Negativity ranking of the situations by experts

**Figure 9:**
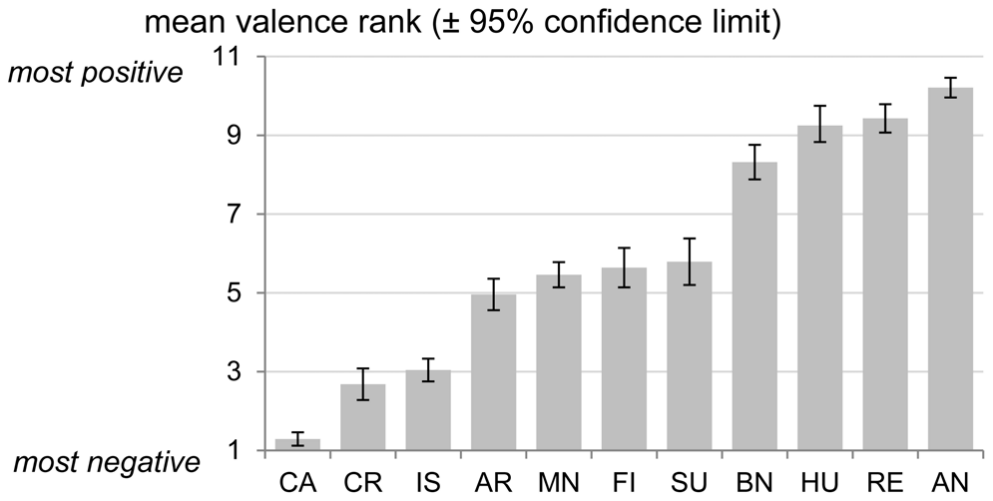
Mean (± confidence interval) rank of valence of the situations as estimated by 28 experts. Situations are ordered by their mean rankings from the most negative to the least negative/most positive.

Mean negativity ranks given by experts to the eleven situations are presented in Figure 9. The experts evaluated castration (mean rank = 1.29) as the most negative situation. Simulated crushing and isolation were also considered as very negative. Holding in arms, missed nursing, fighting and surprise were ranked as moderately negative. Before nursing, huddling and reunion were considered in the least negative (most positive) situations and after nursing (mean rank = 10.21) won the most positive rank.

#### The influence of situation negativity on the acoustic parameters

**Figure 10:**
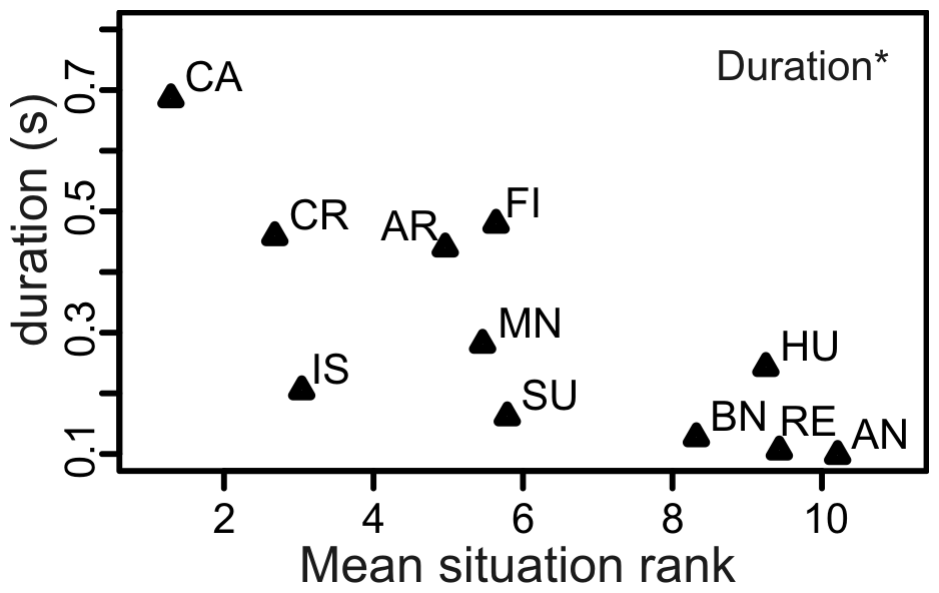
Relationship between the average duration of calls of the 11 situations as a function of the mean valence rank. Valence was evaluated from the point of view of the piglets by experts after correction for multiple comparisons: *: *P<*0.05.

Situations with more negative valence ranking were associated with longer duration calls (Spearman rank correlation: *r*_s_ = -0.80, *N* = 11, adjusted *P* = 0.025, Figure 10). None of the other seven acoustic parameters was associated with the experts’ ranking of situations (*|r_s_|* <0.48, *N* = 11, adjusted *P*>0.45).

#### The influence of situation negativity on the call types

**Figure 11:**
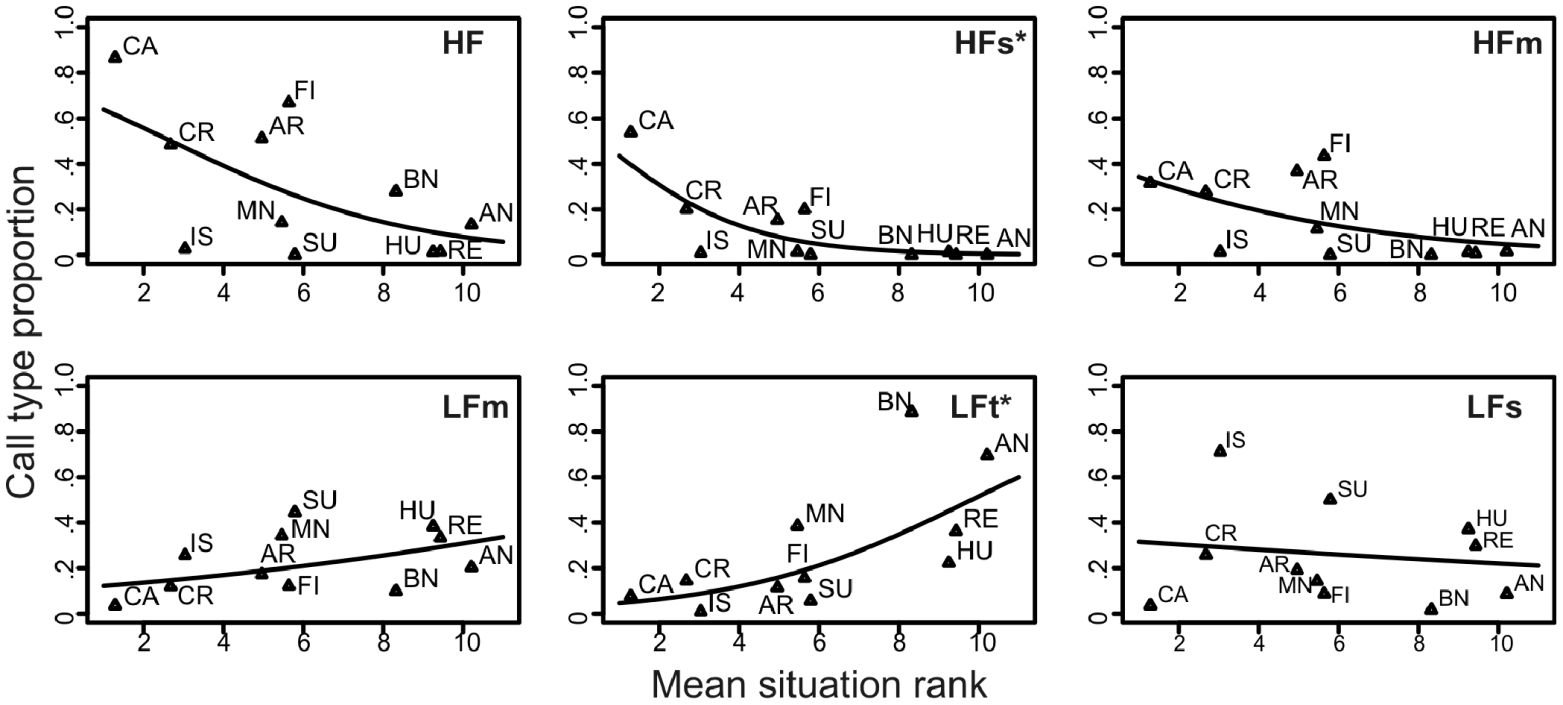
Relationship between the proportion of call types and the valence of the situation. Valence was evaluated from the point of view of the piglets by experts. *t*: 0.05<*P*<0.10;*: *P*<0.05.

The proportions of call types (probabilities that the emitted call would be of a particular call type) were affected by the expert-ranked negativity of the situation (Figure 11). The proportion of high-pitched call types was higher (HFs: *b* = -0.55, *F*_9_ = 15.8, adjusted *P* = 0.012) or tended to be higher in more negative situations (HF: *b* = -0.34, *F*_9_ = 5.9, adjusted *P* = 0.076; HFs: *b* = -0.26, *F*_9_ = 3.8, adjusted *P* = 0.098). The proportion of LFt decreased (*b* = 0.34, *F*_9_ = 14.1, adjusted *P* = 0.012) and the proportion of LFm tended to decrease with negativity of the situation (*b* = 0.13, *F*_9_ = 4.8, adjusted *P* = 0.084). Occurrence of LFs calls was unaffected by the rank (*b* = -0.05, *F*_9_ = 0.2, adjusted *P* = 0.650).

## Discussion

### The Clustered or Graded System

This study is the first systematic description of the vocal repertoire of domestic piglets based on calls recorded in a large variety of contexts. To our knowledge, this is also the first which built a vocal repertoire in an ungulate species based solely on the acoustic properties of the sounds. Indeed, when looking for call types, we did not take into account the context of emission or the function of the calls contrary to many preceding studies in other animals [40]–[42].

A large number (1513) of calls expressed by piglets have been recorded in eleven situations. This allowed us to show that piglets emit a wide variety of sounds from very short to longer than 2s, from low to high frequency and from tonal to noisy. The cluster analysis indicated a relatively clear distinction between two basic types, namely the long high frequency calls and shorter low frequency calls. The classification into five call types was much less distinct, showing that at this level the call types were rather blurred.

The two-call-types and five-call-types clusterings are in agreement with the empirical classifications used in previous pig vocalisation studies. The two-call-types division of the vocal repertoire into low (LF) and high frequency (HF) calls fits with the classifications of previous studies [26], [43], [44] done with calls expressed during castration, weaning and isolation procedures, respectively. In castration for instance, calls are clearly distributed into two categories according to their frequency even if the frequency limits do vary. For instance, in Figure 2 of [43] the authors distinguished high calls with peak frequencies between 3000 and 4000 Hz and low calls with peak frequency between 100 and 600 Hz, while in a more detailed study [29] high calls had a frequency higher than 1000 Hz and low call a frequency lower than 1000 Hz. In the full repertoire recorded in the present study, the multimodal distribution in pitch was also present, but the intermediate calls were also common, probably due to the larger numbers of situations tested. The two clusters can be also distinguished on the basis of the duration of the calls, LF being shorter than HF calls.

The five-call-types solution identified through cluster analysis in the present study divided the calls according to their frequency modulation and entropy (tonality). This reflects the use of mixed call types in the literature [23], [24]. The five-call-types solution shows a good one-to-one mapping to the five call types described previously during nursing interactions [28]. The high, long, tonal HFm calls with strong positive frequency modulation correspond to “screams”. HFs calls with no frequency modulation correspond to “squeaks”. Our moderately tonal and medium pitched LFt calls correspond to “croaking” calls. The atonal low pitched modulated LFm calls correspond to “high” grunts and the stable atonal very low pitched LFs calls correspond to “low” grunts. The five-call-types solution does also map well with the four call types of Kiley [23] disregarding multiple-element calls. “Squeals” clearly encompass both HFs and HFm and “low grunts”, “high grunts” and “chirrups” parallel our LFs, LFm and LFt calls. Another study [17] distinguished only three call types during restraint and castration, their “screams” being our HFm calls, their “squeals” encompassing both HFs and LFt, and their “grunts” corresponding to LFm and LFs. During isolation, squeals and two types of grunts were identified [45], possibly corresponding HF, LFm, LFs, respectively, in our study. The LFm and LFt categories may include “barks” described in piglet startle reactions [18]; however, we had only few Surprise calls in our sample and lack of recordings made during play to conclude.

The close correspondence to previously published vocal repertoires for specific situations strengthens the assertion that piglet vocal repertoire consists of objectively distinct call types. This notion is further supported by the observations that at least squeals and grunts are produced differently. Squeals are emitted orally while the nasal passage is closed by raised velum whereas grunts are emitted orally with velum lowered [46]. On the other hand, the non-distinct courses of the Etak and PreK functions do not allow to come up with an unambiguous “solution” about the number of calls types and therefore about the repertoire size. There is a lot of blurring at the two- and five-call-types level, giving the repertoire a less clear-cut structure than is typical for trully discrete systems such as alarm calls of some monkeys [3], [47], isolation calls of mouse pup calls [48] or bird repertoires [49]. This blurring might be partly explained by the fact that including a large sample of individuals necessarily brings in variation in vocal production organs and production styles leading to variation in the acoustic structure even within the same call type. This may lead to the situation in which the discrete character of the repertoire is blurred as in case of e. g. distinct American English vowels [50]. Also, existing call types may be partly blurred because of ongoing development in piglets during the time period within which the recordings were made. We did not evaluate the effect of age on piglet vocalizations as situations that we recorded were either necessarily (e.g. castration) or by choice (e.g. isolation was recorded at age of one week because it is the age when piglets leave nest and can get lost in natural situations) occurring at certain ages of piglets only. Nevertheless, it is not clear whether the quantitative variation within the call types is mainly a noise due to inter-individual and developmental variability or whether it carries important information about the situation of the animal. In our study, we systematically compared the quantitative analysis of call properties and the qualitative discrimination of call types.

### Vocal Expression in the Different Situations

The eleven situations clearly differed in the acoustic qualities of the emitted calls, thus providing any potential receiver with a rich source of information about the state of the vocalising piglet and the situation. At the same time, calls from situations of similar biological significance were acoustically similar to each other. In life-threatening situations, i.e. Castration, Crushing, Arms and Fighting, piglets produce long, high-pitched tonal calls, mainly of the HFs and HFm types. Consequently the foremost message conveyed seems to be the same, i.e. the gravity of the situation even if the emotional state is different in the four situations, i.e. pain in Castration, pressure in Crushing, psychological distress induced by humans in Arms and distress induced by a littermate in Fighting. However, more precise information may be conveyed leading the mother sow to react appropriately according to the situation, i.e. by maternal aggression against the predator or by standing up in the case of crushing [51] or by interrupting the nursing in the case of severe fighting between piglets [25]. In the nursing situations, the calls were of medium pitch and tonality, belonging mainly to the LFt type. LFt calls may convey identity information towards the sow [20], potentially in the frequency domain only, as in sheep [52] or in time and frequency domains as for sows at the time of nursing [53]. The remaining situations of Reunion, Huddling, Isolation and Surprise produced short, low, noisy calls of types LFm and LFs, although Huddling and especially Reunion also incited some LFt calls typical for mother-directed interactions. The LFm calls and especially the very low and noisy LFs calls could be considered as contact calls, emitted to inform about current location of the piglet, although these calls, too, carry information about individual identity [54]. It is worth noting that especially for the situation with longer durations (castration, crushing, in arms, fighting, huddling, isolation), the proportion of call types may change as the situation continues, due to habituation or fatigue. So our data set contained a representative sample over any time-related changes that may have occurred. The question of how the acoustic output changes with varying duration or severity of a specific situation is very interesting [29] but it was outside the scope of this study.

### How Easy is it to Recognise the Context of Emission from Vocalisations?

Piglet calls differ in acoustic properties between situations but there are overlaps. So we tried, using multi-parametric acoustic analysis, to estimate how easy it could potentially be to identify from a call (or from a small set of calls) in which situation this call/these calls had been emitted. We investigated the accuracy of the interpretation if the full quantitative information about acoustic properties was available or if just the reduced information about call types was available. We also assessed how the potential accuracy is dependent on the number of calls from a situation that are available for the interpretation.

From the quantitative description of one single call with eight parameters, it was possible to identify the exact situation in 28% of the cases. This level can be considered as quite high given the fact that there were 11 situations. The success increased steeply to 60% at 5 calls and reached 90% at 20 calls. Piglets usually emit calls in series which may facilitate recognition of the situation of emission by the receiver. The success in interpreting the calls was even higher if the task was only to distinguish whether the piglet was in a threatening situation, engaged in a nursing, or in another situation (70% of success with a single call). The five cluster solution reached lower situation recognition at 5 calls (45% vs 60%) or at 20 calls (70% vs 90%) than the quantitative perception; this is logical since the 5 call types are a simplification of reality compared to a full description. However, the five call type perception attained the same accuracy as the quantitative perception in two crucial aspects: when only one or two calls were available and if the classification was focused on the gross discernment of the biological significance (life threat/nursing/other).

In brief, our results would suggest that a receiver, who would perceive piglet calls as simply five distinct types and ignore any finer variation, might have enough information to make a correct judgement about the piglet’s basic situation. It remains to be tested if pigs or humans really use the call-types in their evaluation of piglet calls [27], [55], [56], [57]. It is worth noting that the automatic animal call decoding methods that are being developed for pigs [58] and cows [59] are based on the assumption of disjoint call types.

### Link between Vocal Expression and Negativity Ranking

Vocalisations are a promising tool for automatic welfare assessments in many farm animal species [60] based on the assumption that the acoustic quality of the calls is linked to the affective state of the calling animal. Examples of the studies on the effects of emotions on animal vocalizations can be found in [11] and include several studies on pigs [26], [61]. These studies mainly evaluated the effect on their vocalisations of the arousal of the animals, rather than the valence of their emotional state [11]. In the present study, we did not measure the emotional state of the piglets, but asked experts to evaluate the negativity of the situation for the piglets. The experts largely agreed on the different emotional values for the situations, from very negative valence (castration) to very positive valence (after nursing). Even if this negativity ranking is not a proper assessment of the valence (with an independent score), we obtained a relative score between the 11 situations.

Our results suggest that, among the 8 acoustic parameters, only the duration of the call is linked to the valence of the situation for the emitter: longer calls were associated with more negative situations. This is in agreement with results on dogs [10], squirrel monkeys [62] and on ultrasonic vocalisations in rats [63]. Surprisingly, the other seven parameters were not linked to the valence. We could have expected that some of them would have been, like the peak frequency for example [11]. Indeed, all pitch-related parameters showed negative correlations with valence, though non-significant. Interestingly, calls from the three most positive situations did not have the lowest average pitch (contrary to call duration) suggesting that the link between valence and pitch might be U-shaped with both most negative and most positive situations inciting higher-frequency vocalisations (HF and LFt, respectively) than the neutral situations. Many studies found opposite relationship trends between valence and pitch [11]. This might come from the fact that researchers focused on a limited range of situations or call types. However, inspection of our scatterplots did not show clear support for such parabolic distribution of data points.

The proportions of call types were linked or tended to be linked to the valence except for LFs. The long HF calls and especially the HFs type calls were associated with a more negative valence, while shorter and lower pitched LFm and LFt calls corresponded to a less negative valence.

One limitation of our results is that the emotional negativity was assessed by human experts and not obtained from observational studies with the piglets. Indeed, the experts might not have captured the emotional negativity of some situations as it is perceived by the piglets in reality. For instance, isolation was judged as negative as crushing, and much more negative than being held in arms. According to our experience with piglet behaviour and also based on functional evolutionary reasoning, the danger for the piglet is much more serious when it is pressed against the ground (Crushing) or carried away by a “predator” (Arms) than when it loses contact with its mother (Isolation). One reason for the very negative ranking of isolation by the experts might have been that most of them are involved in animal welfare expertise. In this position, they are frequently thinking about isolation in negative terms, both when they argue for group housing of pigs and when they apply isolation as a stressor in their research. Obviously, our results will have to be confirmed by multimodal evaluations of the emotional state of the animals [30], [64]. Studying the effect of the valence of the situation on the vocalisations should also include an assessment of the arousal state, the other component of the emotional state [11], [30].

### Conclusion

The vocal repertoire of piglets is described here for the first time in full through a quantitative acoustic analysis. The analysis indicates that clusters acoustically similar vocal expressions make it possible to identify either two or five call types in the piglet repertoire although theses call types are blurred rather than strictly discrete. The five call type description corresponds well to previously published partial repertoires in specific situations. Clear links exist between the type of situation and the vocal expression in that situation, each situation being identifiable by the vocal expression. These links can be adequately described both with a set of quantitative acoustic variables and through the categorisation into call types.

## Supporting Information

Table S1 **Call parameters measured for each call.**
(DOCX)

Table S2 **Mean ± standard error of acoustic parameters for each cluster.**
(DOCX)

Table S3 **Results of discriminant analyses with call clusters as grouping variable.** A. 2-cluster solution and B. 5-cluster solution.
(DOCX)
