# Supplementary material for: Encoding of Situations in the Vocal Repertoire of Piglets (Sus scrofa): A Comparison of Discrete and Graded Classifications
Source: PLoS One. 2013 Aug 13;8(8):e71841. doi: 10.1371/journal.pone.0071841 (PMC3742501; doi:10.1371/journal.pone.0071841)
Supplement: Table S1 — Call parameters measured for each call. (DOCX) [file pone.0071841.s001.docx]

Table S1

| variable | definition | |
| --- | --- | --- |
|  |  | |
| Spectral measures (measured from spectrum of entire call) | | |
| pf (Hz)*^a^* | peak frequency; frequency with the highest amplitude | |
| q25 (Hz)^b^ | frequency equivalent to 25 quartile of distribution of frequency amplitudes (high correlation with q50) | |
| q50 (Hz)*^a^* | frequency equivalent to 50 quartile of distribution of frequency amplitudes | |
| q75 (Hz)^b^ | frequency equivalent to 75 quartile of distribution of frequency amplitudes (high correlation with q50) | |
|  |  | |
| Tonal quality of the call (measured from averaged spectrum of entire call) | | |
| ent*^a^* | mean Weiner entropy of the call; ratio of the geometric mean to the arithmetic mean of the spectrum; as Weiner entropy intrinsically increases with bandwidth of the sound (when keeping the sampling frequency constant), we scaled the value reported by SASLab to q50. A low entropy corresponds to a tonal sound, while a high entropy corresponds to a noisy sound. | |
| hnr (dB) | mean harmonic to noise ratio; the noise floor of the spectrum is estimated by calculating the moving average of the spectrum; the maximum distance between the original spectrum and the filtered spectrum (expressed in dB) is taken as the HNR measure | |
| peaks | mean number of harmonic peaks above -20 dB relative to maximum amplitude; with 30 dB peak hysteresis; this setting has been used as it resulted for satisfying detection of peaks judged by eye inspection of spectrograms | |
|  |  | |
| Frequency modulation (calculated from 9 measurement points spaced equidistantly within a call; call was divided into 10 segments of the same length resulting in 11 measurement points; the first and the last point were excluded) | | |
| q50start (Hz)*^a^* | | q50 at the beginning of the call (the first of 9 measurement points) |
| q50end (Hz)*^a^* | | q50 at the end of the call (the last of 9 measurement points) |
| q50max (Hz)^b^ | | maximum value of q50 within the call (over 9 measurement points ; high correlation with q50) |
| q50min (Hz)*^a^* | | minimum value of q50 within the call (over 9 measurement points) |
| q50range (Hz) | | difference between minimum and maximum q50 values |
| q50linREL | | linear slope of q50 within the call; relative position of q50 samples within a call has been used because when using absolute times for regression, short calls intrinsically have much higher regression coefficients to such an extent that call duration is the main factor determining the slope; using relative measures stresses the shape over duration |
| q50quadREL | | second order polynomial coefficient of q50 within the call with relative timing used for calculation of the coefficient as in q50linREL |
| q50change (Hz) | | mean difference (absolute value) in q50 between two successive measurement points within the call |
| q50SD (Hz) | | standard deviation of q50 within the call |
|  | |  |
| Amplitude modulation (calculated from nine, regularly spaced, measurement points over the call) | | |
| pamin (dB) | | minimum peak amplitude (pa) scaled to maximum peak amplitude within the call |
| parange (dB) | | difference between minimum and maximum peak value relative to maximum pa |
| palinREL | | linear slope of pa within the call; relative position instead of absolute time values were taken as x-values and amplitudes relative to maximum amplitude within the call were taken as y-values |
| paquadREL | | second order polynomial coefficient of pa within the call; relative position instead of absolute time values were taken as x-values and amplitudes relative to maximum amplitude within the call were taken as y-values |
| pachange (dB) | | mean difference (absolute value) in pa relative to maximum between two successive measurement points within the call |
| paSD (dB) | | standard deviation of pa within the call |
|  | |  |
| Temporal measures | | |
| dur (s)*^a^* | duration | |
| q50maxloc*^a^* | relative location of q50max within the call | |
| q50minloc | relative location of q50min within the call | |
| paminloc | relative location of minimum pa | |
| pamaxloc | relative location of maximum pa | |

^a^ bimodal or multimodal variables that were used for the cluster analyses

^b^ bimodal or multimodal variables that were not used for the cluster analyses due to high correlation with some of the other variables that were used for cluster analysis (correlation indicated in parentheses). See main text for selection procedure.
