# Supplementary material for: Encoding of Situations in the Vocal Repertoire of Piglets (Sus scrofa): A Comparison of Discrete and Graded Classifications
Source: PLoS One. 2013 Aug 13;8(8):e71841. doi: 10.1371/journal.pone.0071841 (PMC3742501; doi:10.1371/journal.pone.0071841)
Supplement: Table S2 — Mean ± standard error of acoustic parameters for each cluster. (DOCX) [file pone.0071841.s002.docx]

Table S2

|  | 2-cluster solution | | | | | | |  |  | 5-cluster solution | | | |  |  |  |  |  |  |  |  |  |  |  |  |  |  |  |
| --- | --- | --- | --- | --- | --- | --- | --- | --- | --- | --- | --- | --- | --- | --- | --- | --- | --- | --- | --- | --- | --- | --- | --- | --- | --- | --- | --- | --- |
|  | **1 - LF** |  |  |  | **2 - HF** | | |  |  | **1 - LFm** | |  |  | **2 - LFt** | |  |  | **3 - LFs** | |  |  | **4 - HFs** | |  |  | **5 - HFm** | |  |
| **duration (s)** | 0.19 | ± | 0.14 |  | 0.60 | ± | 0.35 |  |  | 0.18 | ± | 0.13 |  | 0.15 | ± | 0.11 |  | 0.20 | ± | 0.12 |  | 0.77 | ± | 0.37 |  | 0.58 | ± | 0.27 |
| **pf (Hz)** | 666 | ± | 558 |  | 3005 | ± | 1319 |  |  | 532 | ± | 372 |  | 1307 | ± | 737 |  | 354 | ± | 181 |  | 3782 | ± | 1222 |  | 2513 | ± | 1201 |
| **q50 (Hz)** | 1268 | ± | 746 |  | 4079 | ± | 1075 |  |  | 1103 | ± | 533 |  | 2283 | ± | 627 |  | 681 | ± | 252 |  | 4915 | ± | 909 |  | 3541 | ± | 832 |
| **ent** | 0.299 | ± | 0.096 |  | 0.128 | ± | 0.030 |  |  | 0.315 | ± | 0.080 |  | 0.185 | ± | 0.037 |  | 0.374 | ± | 0.055 |  | 0.109 | ± | 0.020 |  | 0.144 | ± | 0.034 |
| **q50start (Hz)** | 1202 | ± | 765 |  | 3130 | ± | 1434 |  |  | 883 | ± | 377 |  | 2253 | ± | 943 |  | 825 | ± | 561 |  | 4222 | ± | 1278 |  | 2053 | ± | 798 |
| **q50end (Hz)** | 1315 | ± | 903 |  | 4265 | ± | 1375 |  |  | 1492 | ± | 998 |  | 1998 | ± | 639 |  | 637 | ± | 265 |  | 4959 | ± | 1212 |  | 4067 | ± | 1265 |
| **q50min (Hz)** | 895 | ± | 500 |  | 2609 | ± | 1107 |  |  | 756 | ± | 322 |  | 1612 | ± | 469 |  | 534 | ± | 156 |  | 3645 | ± | 820 |  | 1790 | ± | 708 |
| **q50maxloc** | 4.5 | ± | 2.8 |  | 5.7 | ± | 2.6 |  |  | 7.6 | ± | 1.4 |  | 3.8 | ± | 2.2 |  | 2.2 | ± | 1.3 |  | 4.9 | ± | 2.5 |  | 7.1 | ± | 1.8 |
