# Supplementary material for: Encoding of Situations in the Vocal Repertoire of Piglets (Sus scrofa): A Comparison of Discrete and Graded Classifications
Source: PLoS One. 2013 Aug 13;8(8):e71841. doi: 10.1371/journal.pone.0071841 (PMC3742501; doi:10.1371/journal.pone.0071841)
Supplement: Table S3 — Results of discriminant analyses with call clusters as grouping variable. A. 2-cluster solution and B. 5-cluster solution. (DOCX) [file pone.0071841.s003.docx]

Table S3

| **A 2-cluster solution** | |  |  |  |  |  |
| --- | --- | --- | --- | --- | --- | --- |
|  | **standardized coefficients** | |  | **unstandardized coefficients** | | |
|  | **LD1** |  |  | **LD1** |  |  |
| **duration** | -0.33 |  |  | -1.39669 |  |  |
| **pf** | -0.47 |  |  | -0.00058 |  |  |
| **q50** | -0.57 |  |  | -0.00067 |  |  |
| **ent** | -0.22 |  |  | -2.64798 |  |  |
| **q50start** | -0.23 |  |  | -0.00023 |  |  |
| **q50end** | -0.30 |  |  | -0.00028 |  |  |
| **q50min** | 0.27 |  |  | 0.00037 |  |  |
| **q50maxloc** | 0.00 |  |  | 0.00069 |  |  |
|  |  |  |  |  |  |  |
|  |  |  |  |  |  |  |
| **B 5-cluster solution** | |  |  |  |  |  |
|  | **standardized coefficients** | |  | **unstandardized coefficients** | | |
|  | **LD1** | **LD2** |  | **LD1** | **LD2** |  |
| **duration** | 0.34 | -0.15 |  | 1.68556 | -0.73241 |  |
| **pf** | 0.37 | -0.01 |  | 0.00051 | -0.00002 |  |
| **q50** | 0.43 | -0.24 |  | 0.00071 | -0.00040 |  |
| **ent** | 0.00 | -0.65 |  | -0.07281 | -12.38772 |  |
| **q50start** | 0.11 | 0.17 |  | 0.00014 | 0.00022 |  |
| **q50end** | 0.28 | -0.10 |  | 0.00033 | -0.00012 |  |
| **q50min** | 0.28 | -0.41 |  | 0.00059 | -0.00086 |  |
| **q50maxloc** | 0.14 | 0.87 |  | 0.07455 | 0.47738 |  |
| **proportions of explained variance: LD1 = 0.78, LD2 = 0.11** | | | | |  |  |
